# Naringenin mitigates autoimmune features in lupus-prone mice by modulation of T-cell subsets and cytokines profile

**DOI:** 10.1371/journal.pone.0233138

**Published:** 2020-05-18

**Authors:** Amayrani Abrego-Peredo, Héctor Romero-Ramírez, Enrique Espinosa, Gabriela López-Herrera, Fabio García-García, Mónica Flores-Muñoz, Claudia Sandoval-Montes, Juan Carlos Rodríguez-Alba

**Affiliations:** 1 Programa de Doctorado en Ciencias de la Salud, Instituto de Ciencias de la Salud, Universidad Veracruzana, Xalapa, Veracruz, México; 2 Unidad de Citometría de Flujo, Instituto de Ciencias de la Salud, Universidad Veracruzana, Xalapa, Veracruz, Mexico; 3 Departamento de Biomedicina Molecular, Centro de Investigación y de Estudios Avanzados del IPN, Ciudad de México, México; 4 Investigación en Inmunología Integrativa, Instituto Nacional de Enfermedades Respiratorias, Ciudad de México, México; 5 Unidad de Investigación en Inmunodeficiencias, Instituto Nacional de Pediatría, Ciudad de México, México; 6 Laboratorio de Biología del Sueño, Instituto de Ciencias de la Salud, Universidad Veracruzana, Xalapa, Veracruz, México; 7 Laboratorio de Investigación en Medicina Traslacional, Instituto de Ciencias de la Salud, Universidad Veracruzana, Xalapa, Veracruz, México; 8 Departamento de Inmunología, Escuela Nacional de Ciencias Biológicas, Instituto Politécnico Nacional, Ciudad de México, México; Instituto Nacional de Ciencias Medicas y Nutricion Salvador Zubiran, MEXICO

## Abstract

Naringenin is flavonoid mainly found in citrus fruits which has shown several biological properties. In this work, we evaluated the therapeutic potential of the flavonoid Naringenin. Five-month-old B6.MRL-Fas^lpr^/J lupus-prone mice were administered daily orally with Naringenin for seven months. We showed that Naringenin treatment at 50 or 100 mg/kg inhibited the splenomegaly and decreased the levels of anti-nuclear and anti-dsDNA autoantibodies. Furthermore, a reduction in serum concentration of TNF-α, IFN-γ and IL-6 was observed in the mice provided with Naringenin. Interestingly, serum levels of IL-10 increased. Naringenin decreased the frequency and absolute numbers of splenic effector memory T cells. Additionally, in order to be able to evaluate whether Naringenin prevented kidney damage, twelve-week-old MRL/MpJ-Fas^lpr^/J mice, an accelerated lupus model, were orally administered with Naringenin at 100 mg/kg for six weeks. Surprisingly, Naringenin treatment prevented kidney damage and reduced the development of fibrosis similar to cyclophosphamide group. Moreover, Naringenin treatment increased the percentage of regulatory T cells in this aggressive model of lupus. Together, these results suggest a potential ability of Naringenin to reduce the autoimmunity in lupus-prone mice by modulation of T-cell subsets and cytokines profile that mitigate the development of important lupus clinical manifestations.

## Introduction

Systemic Lupus Erythematosus (SLE) is a chronic disease that is known to predominantly affect women (**~**9:1 with respect to men). The incidence and prevalence of SLE continues to increase, while being most frequent in African, Asian and Hispanic populations [1,2]. SLE is triggered by the interaction of genetic and environmental factors that lead to the loss of immunological tolerance. The main hallmark of SLE is the production of high amounts of autoantibodies responsible for tissue damage [3–5]. Additionally, a spontaneous activation of T cells occurs which leads to the expansion of CD4^+^CD44^+^CD62L^-^ T effector memory cells [6–8]. Once activated, T cells increase their production of proinflammatory cytokines such TNF-α, IFN-γ and IL-6 [9–11]. Moreover, regulatory T cells (Tregs) are important in suppression of immune response and prevent autoimmune disorders through different mechanisms including the production of anti-inflammatory cytokines (IL-10, TGF-β) or cell-cell contact [12,13]. Until now, there is controversial data about the proportion of Tregs in lupus. Some reports suggest that SLE patients had decreased numbers of Tregs in peripheral blood. Conversely, other authors have reported unaltered or increased proportions of Tregs. In addition, the information about suppression activity of Tregs in SLE is inconclusive, while some studies show a reduction in their suppressive function, others did not observe functional deficiencies [14–16]. Other important fact is that effector cells have a reduced sensitivity to suppression by Tregs [17]. On the other hand, B-cell hyperactivity is enhanced by T cells, leading to the constant production of autoantibodies, furthermore, B cells take up autoantigens and acting as antigen-presenting cells to T cells [18,19].

Conventional therapies for SLE combine antimalarial, steroidal, nonsteroidal drugs and immunosuppressive agents including: cyclophosphamide, azathioprine and mycophenolate mofetil. Despite that, these drugs improve survival and provide a better lifespan for patients, all of them produce adverse effects [20]. Currently, biological therapies use monoclonal antibodies to block important proteins involved with the activation and effector functions of different lymphocytes. Nevertheless, this strategy has some restrictions and its effectiveness is not clear [18,21]. Therefore, it is necessary to search for new therapeutic strategies. In fact, the study of flavonoids in the treatment of inflammatory and autoimmune diseases has increased in last decade [22–24].

Naringenin (5,7,4´-trihydroxyflavanone) is a flavonoid mainly found in citrus fruits, predominantly in grapefruits and oranges, which has shown to have different biological effects including anti-inflammatory and immune modulatory activities [22,25,26]. In this regard, Naringenin has shown to reduces mRNA expression, production and release of pro-inflammatory cytokines during inflammatory response models *in vivo* and *in vitro* [27–29]. Recent studies have demonstrated that Naringenin decreases the percentages of proinflammatory T-cell subsets in experimental autoimmune encephalomyelitis (EAE) [30–32]. Interestingly, Naringenin has also shown to increase the percentage of Tregs and improve their suppressive function in an inflammatory model [33]. Together, these data suggest the therapeutic potential of Naringenin in the treatment of immunological disorders.

Additionally, it has been reported that the oral supplementation of flavonoids such as Astilbin or Baicalin in MRL/MpJ-Fas^lpr^/J mice, a lupus-prone model, improve the severity of the main autoimmune manifestations. This effect results from the ability of flavonoids to regulate the imbalance between different T lymphocytes subsets [34,35]. Moreover, it has reported that *in vitro* several flavonoids increased the percentage of Tregs and their suppressive activity in human PBMCs from healthy donors and SLE patients [36,37]. However, the potential therapeutic effect of Naringenin on SLE has not been characterized either *in vitro* or *in vivo*. Thus, the aim of this work was to evaluate the effect of oral administration of Naringenin in lupus-prone mice.

## Materials and methods

### Mice

Male B6.MRL-Fas^lpr^/J, MRL/MpJ-Fas^lpr^/J and C57BL/6J (wild-type mice) mice were purchased from The Jackson Laboratory (Bar Harbor, Maine, USA). The mice were bred by strain to obtain littermates in the Universidad Veracruzana animal facility according to national regulations (NOM-062-ZOO-1999). Moreover, mice were maintained with cardboard tube for environmental enrichment. Five-month-old B6.MRL-Fas^lpr^/J and C57BL/6J mice were used for experiments. In some cases, we used twelve-week-old MRL/MpJ-Fas^lpr^/J mice. The mice were sacrificed by cervical dislocation. All procedures were approved by the Institutional Animal Care and Use Committee (IACUC) of the Health Sciences Institute (Registration number: 2017–0011).

### Naringenin treatment

Five-month-old B6.MRL-Fas^lpr^/J mice were orally administered by gavage (esophageal stainless steel cannula, 20G X1.5, Cadence Science®, Mexico) with Naringenin (≥ 95% purity, Sigma-Aldrich, Toluca, Mexico) dissolved in 0.5% carboxymethyl cellulose (Sigma, Aldrich, Toluca, Mexico) in the concentration of 50 (n = 5) or 100 mg/kg (n = 8) daily for seven months. The vehicle group was administered in an equivalent volume (compared with Naringenin at 100 mg/kg) of 0.5% of carboxymethyl cellulose (n = 5 mice). Additionally, as a positive control, the conventional drug to treat SLE, cyclophosphamide (PiSA, Mexico) was injected intraperitoneally at a dose of 20 mg/kg every six days (n = 5 mice). Furthermore, five-month-old C57BL/6J mice were orally administered by gavage with 100 mg/kg of Naringenin or with vehicle for seven months (n = 5 mice).

As the lupus nephritis is less evident in B6.MRL-Fas^lpr^/J mice compare with MRL/MpJ-Fas^lpr^/J mice [38], to evaluate kidney damage, in some experiments, twelve-week-old MRL/MpJ-Fas^lpr^/J mice were administered orally by gavage with Naringenin 100 mg/kg or vehicle for six weeks. Also, one group was intraperitoneally injected with cyclophosphamide 20 mg/mg every six days (n = 5 mice in each group).

### Proteinuria

Proteinuria was measured every month. Urine protein levels were semi-quantitative evaluated using Uristix® 4 test strips (Bayer, Mexico). Proteinuria scores were interpreted as described by Jiang *et al*. [39]: 0, negative; 1, trace; 2 (30 mg/d); 3, (100 mg/d); 4, (300 mg/d) and 5 (2000 mg/d or more).

### Anti-nuclear antibodies

To obtain serum, before sacrificing the mice we bled each one by puncturing of the submandibular vein [40]. Following that, anti-nuclear autoantibodies (ANAs) were determined by indirect immunofluorescence assay. Each serum sample at 1:50 dilution was added to Hep-20 slides (EUROIMMUN, Lübeck, Germany). Slides were incubated at room temperature in a moist chamber for 30 minutes. Subsequently, they were stained with FITC-conjugated goat anti-mouse IgG (Kirkegaard & Perry Laboratories, Maryland, USA) in a dilution of 1:500 (1mg/mL) for 30 minutes. Slides were visualized using an Eclipse epifluorescence microscope (Nikon, Tokyo, Japan) and images were analyzed with ImageJ software (NIH, Bethesda, Maryland).

### Anti-dsDNA IgG detection

Serum titers of anti-dsDNA antibodies were measured by ELISA assay. Previously, plasmid pUC19 was obtained by bacterial transformation and purified by AxyPrep Plasmid Maxiprep Kit® (AXYGEN Biosciences, Union City, USA) under the manufacturer´s instructions. 96-well plates were coated with 20 ng/well of linearized pUC19 plasmid and incubated in a carbonate buffer (pH 9.5) overnight at 4°C. The plates were incubated with blocking buffer (2% fetal bovine serum, 0.05% Tween in PBS). Serial dilutions of serum were added and incubated at room temperature for two hours. HRP-conjugated anti-mouse IgG (Jackson InmunoResearch, West Grove, USA) was added at a 1:3000 dilution. Ten minutes after adding tetramethylbenzidine substrate, the reaction was stopped with 1M phosphoric acid (Sigma-Aldrich). Absorbance was measured at 450 nm using a ELISA reader (BioTec´s EpochTM Multi-Volume Specrophotometer System).

### Serum cytokine levels

Serum concentrations of TNF-α, IFN-γ, IL-6 and IL-10 was evaluated by Mouse ELISA MAX^TM^ DELUXE Set (Biolegend, San Diego, CA) according to the manufacturer’s protocol.

### Immunophenotyping of T- cell subsets by flow cytometry

Spleen cell suspensions from each mouse were obtained by dissociation of tissue and red blood cells were lysed with 0.85% ammonium chloride solution. To analyze T cells subsets, one million splenocytes were stained for 15 minutes with the following antibodies: anti-CD3-BV605, anti-CD4-PerCP-Cy5.5, anti-CD44-PE-Cy7 and anti-CD62L-APC (BD Biosciences). After incubation, the cells were washed with PBS and fixed with 1% formaldehyde in PBS. To analyze Treg cells, 1X10^6^ splenocytes were stained with anti-CD3-BV421, anti-CD4-APC and anti-CD25-APC-Cy7. To detect intracellular FoxP3 expression, cells were permeabilized (Cytofix/Cytoperm TM Fixation/Permeabilization Solution Kit, BD Biosciences) and anti-FoxP3-Alexa488 was added and incubated for 30 minutes. Data were acquired in BD LRS Fortessa flow cytometer (Becton-Dickinson, San Jose, CA) and analyzed by FlowJo software (FlowJo, Tree Star, Ashland, OR).

### Renal damage determination

The kidneys were removed from each mouse and maintained in 10% formaldehyde. Subsequently, they were progressively dehydrated, embedded in paraffin and sectioned to 5 μm thickness. Hematoxylin and Eosin (H&E) and Picrosirius Red (PSR) staining were used for histological assessment. The severity of kidney damage and tissue collagen deposition were analyzed by an expert pathologist without information about the mice and their conditions (blinded assay). To quantify fibrosis Image-ProPlus software (Media Cybernetics, Basingtoke, UK) was used. The area for quantification was selected using Area of Interest Macro and Pixel values were transformed to optical density.

### Statistical analysis

Data was analyzed by one or two-way analysis of variance (ANOVA). Statistical significance was considered when the *p* value was ≤ 0.05. Tukey or Dunnett´s post-hoc test were used for multiple comparison as indicated. All data are expressed as mean ± SD.

## Results

### Naringenin treatment ameliorated the splenomegaly in B6.MRL-Fas^lpr^/J mice

**Fig 1:**
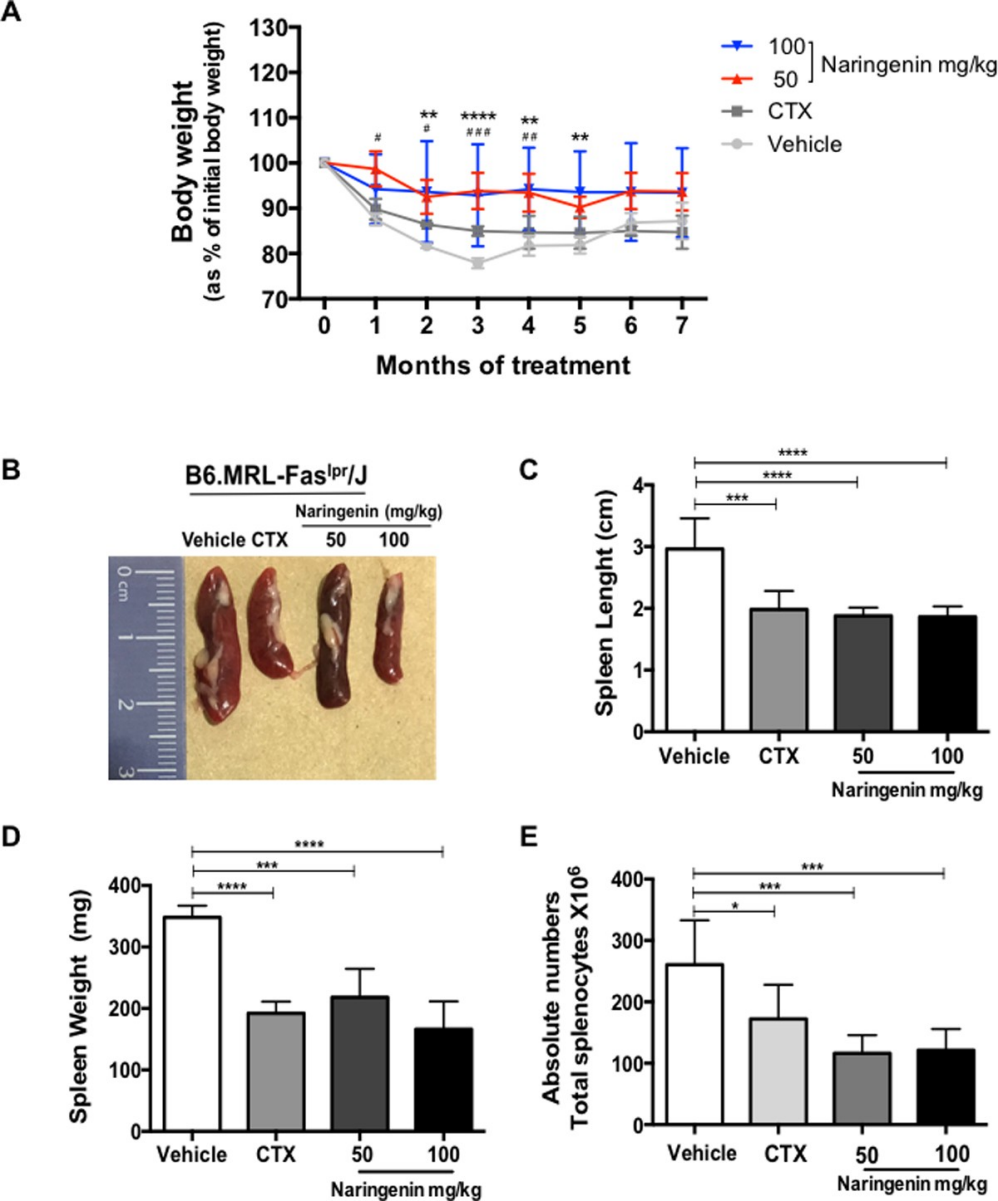
Naringenin ameliorated the enlarged spleen in B6.MRL-Fas^lpr^/J mice. Five-month-old B6.MRL-Fas^lpr^/J mice were oral administered by gavage daily with Naringenin at 50 or 100 mg/kg for seven months. (A) Naringenin treatment improved the body weight loss. (B) Naringenin oral administration prevented the development of splenomegaly. (C) The length and (D) weight of the spleens from mice treated with Naringenin was reduced. (E) The mice provided with Naringenin showed a reduction in the total splenocytes number compared with vehicle group. Statistics was performed using a one-way (B, C, D, E) or two-way (A) ANOVA followed by Tukey’s test. Data presented as mean ± SD, n ≥ 5 mice. Naringenin 100 mg/kg: * p ≤ 0.05, ** p ≤ 0.01, *** p ≤ 0.001, **** p ≤ 0.0001 versus vehicle; Naringenin 50 mg/kg: # p ≤ 0.05, ## p ≤ 0.01, ### p ≤ 0.001 versus vehicle.

To evaluate the therapeutic potential of Naringenin on SLE, five-month-old B6.MRL-Fas^lpr^/J mice were orally administered by gavage with 50 or 100 mg/kg daily for seven months and compared with vehicle and cyclophosphamide groups. The mice in all groups showed a progressive weight loss. Interestingly, the percentage of body weight loss was significantly ameliorated when B6.MRL-Fas^lpr^/J mice were provided with both dose of Naringenin since the first month of treatment. However, by the sixth month of treatment, all mice exhibited similar body weight (Fig 1A). In addition, Naringenin oral administration did not induce a change in the percentage of body weight in the wild type mice (S1A Fig). The spleen enlargement was significant in the B6.MRL-Fas^lpr^/J mice from vehicle group, whereas it was ameliorated in the groups administered with the flavonoid (Fig 1B and 1D). Naringenin treatments showed a significant reduction of the splenomegaly compared with vehicle group. Furthermore, Naringenin treatment did not affect the physical features of the spleen in C57BL/6J mice (S1B–S1D Fig). Additionally, both concentrations of Naringenin significantly diminished the total splenocytes numbers compared with vehicle group (Fig 1E). By other hand, the wild type mice under flavonoid treatment did not show differences in the total splenocytes numbers (S1E Fig).

### Naringenin mitigated the manifestations associated with autoimmunity in B6.MRL-Fas^lpr^/J mice

**Fig 2:**
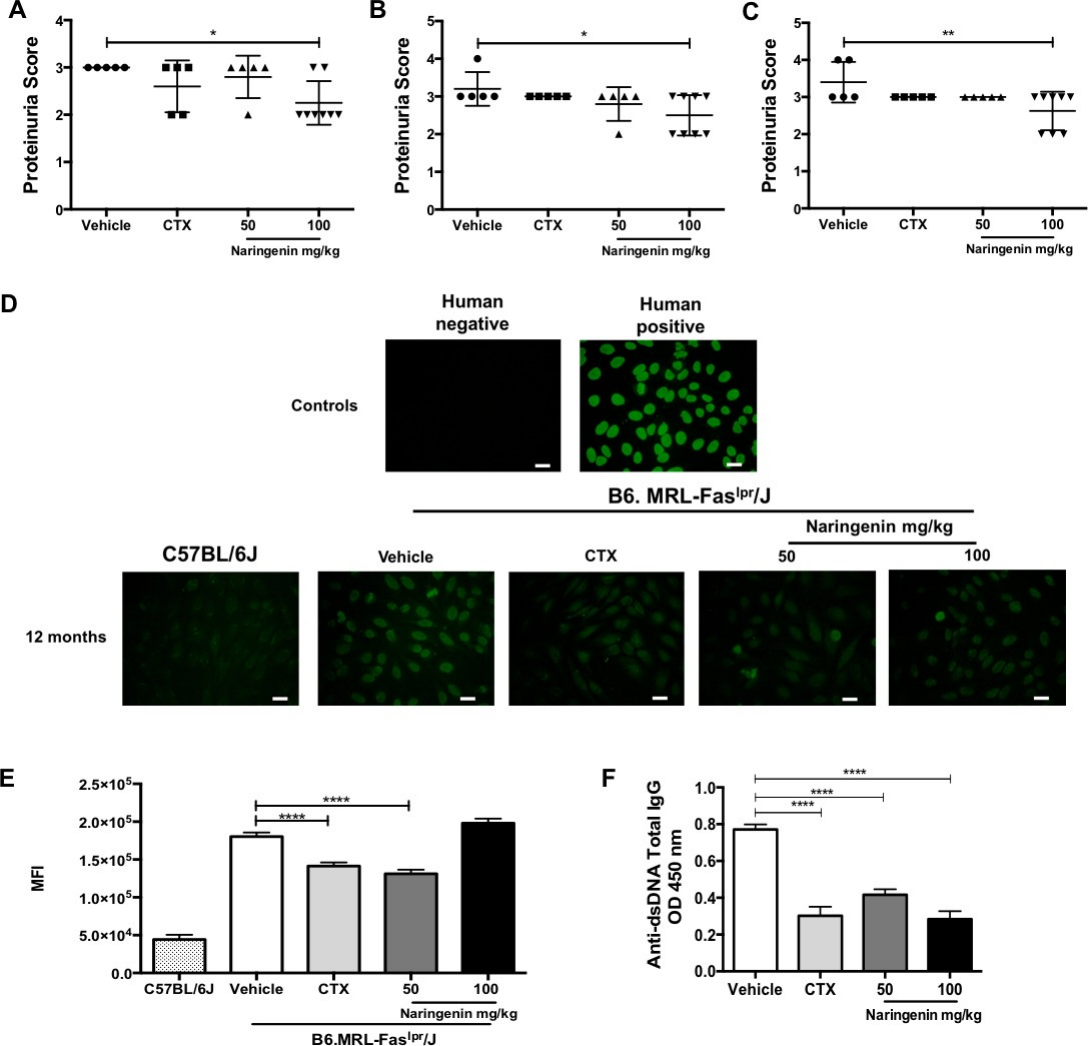
Naringenin alleviates the main signs of SLE in B6.MRL-Fas^lpr^/J mice. Proteinuria was semi-quantitative evaluated by test strips every month. (A-C) The mice provided with the highest dose of Naringenin treatment showed a reduction in the proteinuria score from the first to third month. (D) ANA IgG was determinate using 1:50 from each serum sample. Staining was performed with goat anti-mouse IgG FITC and observed in Eclipse epifluorescence microscope. The negative and positive controls were provided by the manufacture (conjugate anti-human IgG, FITC-labelled). Bars, 25 μm. Naringenin treatment at 50 mg/kg diminished the presence of ANAs. (E) Mean fluorescence intensity of ANAs IgG. Analysis was performed using ImageJ software. (F) Serum concentration of anti-dsDNA was measured by ELISA assay. Naringenin treatments reduced significantly the titers of anti-dsDNA. Statistics was determinate by one-way ANOVA followed by Dunnett (A-B) and Tukey’s test (E-F). Data presented as mean ± SD, n≥5 mice. *p≤ 0.05, ** p≤ 0.01, ***p≤ 0.001, **** p≤ 0.0001 versus vehicle.

Proteinuria is one of the main biomarkers to measure glomerulonephritis, progression and prognosis of the SLE [41,42]. Urine protein levels were measured monthly to determinate kidney involvement. At five months old, when the B6.MRL-Fas^lpr^/J mice began to be administered with Naringenin, all groups had a proteinuria equal to 2 (S2A Fig). Proteinuria score ≥ 2 means that the disease is already established. Fig 2A–2C shows that B6.MRL-Fas^lpr^/J mice from vehicle group had a higher proteinuria score from the first to the third month of treatment, while B6.MRL-Fas^lpr^/J mice treated with Naringenin at 100 mg/kg had a statistically significant reduction in the progression of proteinuria. On the other hand, a slight reduction in the proteinuria was also observed in the B6.MRL-Fas^lpr^/J were injected intraperitoneally with cyclophosphamide, without observing significant differences. From the fourth month of treatment there were no statistically significant differences in the proteinuria score between groups (S2B–S2E Fig).

ANAs and anti-dsDNA are considered to be hallmarks of SLE and they take important role in the pathogenesis [43]. In this regard, we measured the serum concentration of ANAs and anti-dsDNA IgG by indirect immunofluorescence and ELISA assay, respectively. The results showed a significant reduction of ANAs in the B6.MRL-Fas^lpr^/J mice treated with 50 mg/kg Naringenin compared with mice that were only provided with vehicle. Surprisingly, higher dose of Naringenin did not show significant differences respect to vehicle group (Fig 2D and 2E). As expected, B6.MRL-Fas^lpr^/J mice from vehicle group had high levels of anti-dsDNA IgG antibodies in serum. Instead, both doses of Naringenin induced a significant reduction in serum anti-dsDNA IgG antibody levels (Fig 2F). In fact, the levels of serum anti-dsDNA IgG from B6.MRL-Fas^lpr^/J mice administered with the highest dose of Naringenin or cyclophosphamide were similar.

### Naringenin changed the serum concentration of proinflammatory and anti-inflammatory cytokines

**Fig 3:**
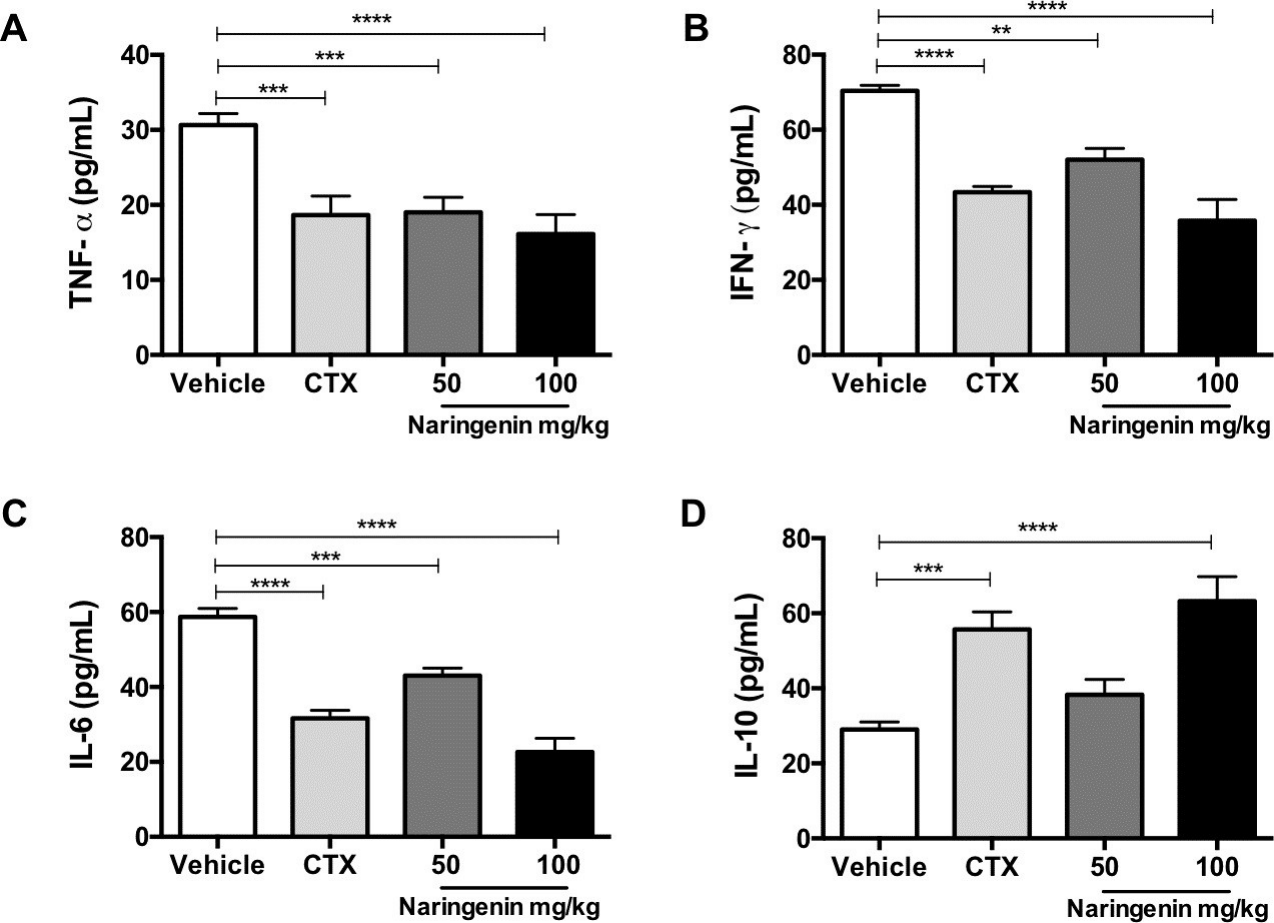
Naringenin reduced the serum concentration of proinflammatory cytokines and increased the concentration of IL-10. Previous to sacrifice, serum was obtained from each mouse by blood sampling of the submandibular vein. The cytokines concentration from each serum sample was measured by ELISA assay. Naringenin reduced the serum concentration of proinflammatory cytokines as (A) TNF-α, (B) IFN- γ, (C) IL-6. Conversely, Naringenin at a dose of 100 mg/kg significantly increased the serum concentration of (D) IL-10, an anti-inflammatory cytokine. Statistical analysis was carried out by one-way ANOVA followed by Tukey’s test and data presented as mean ± SD, n ≥ 5. ** p≤ 0.01, *** p ≤ 0.001, **** p ≤ 0.0001 compared versus vehicle.

Changes in the serum concentration of several cytokines is associated with the disease activity in SLE patients [44]. For this reason, serum levels of TNF-α, IFN-©, IL-6 and IL-10 from B6.MRL-Fas^lpr^/J mice undergoing treatments were measured by ELISA assay. Our results demonstrated that the B6.MRL-Fas^lpr^/J mice, administered daily with Naringenin 50 mg/kg, significantly reduced the serum concentration of TNF-α at similar levels than cyclophosphamide therapy, whereas the B6.MRL-Fas^lpr^/J mice provided with the highest dose of Naringenin, showed a slightly higher reduction (Fig 3A). Furthermore, Naringenin treatment produced a reduction in the serum levels of IFN-© and IL-6 in a dose-dependent manner. Indeed, the higher dose of Naringenin showed a better effect in the IL-6 concentration compared with the cyclophosphamide (Fig 3B and 3C). Interestingly, serum concentration of IL-10 was remarkably increased by Naringenin administration at a dose of 100 mg/kg. Nevertheless, the lower dose of Naringenin did not produce a significant change in the IL-10 levels compared with vehicle group (Fig 3D).

### Naringenin treatment decreases CD4^+^CD44^hi^CD62L^-^ effector memory T-cell subset in B6.MRL-Fas^lpr^/J mice

**Fig 4:**
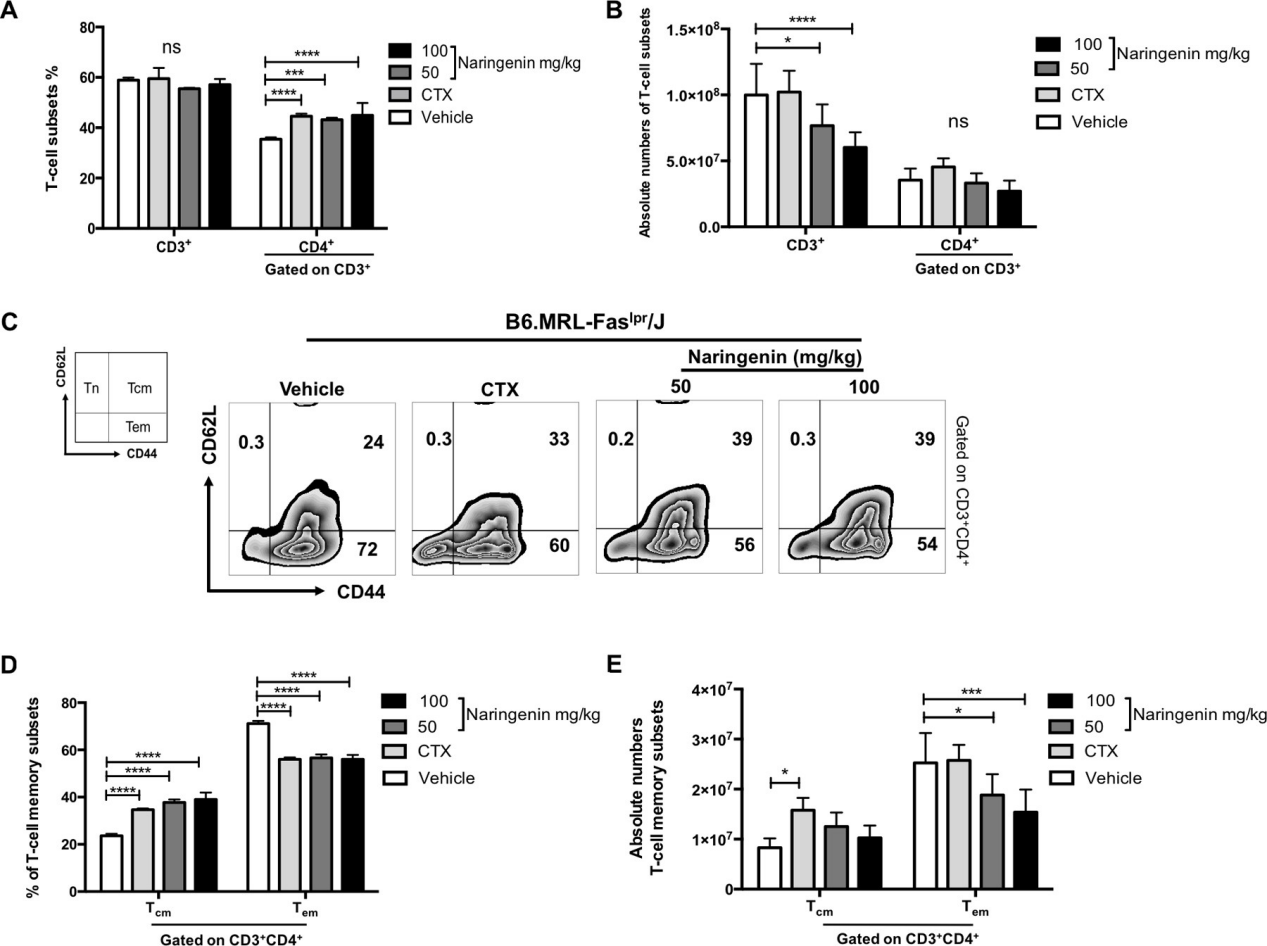
Naringenin treatment diminished splenic CD4^+^CD44^hi^CD62L^-^ effector memory cells in B6.MRL-Fas^lpr^/J mice. Total splenic cells were stained with CD3-BV605, anti-CD4-PerCP-Cy5.5, anti-CD44-PE-Cy7 and anti-CD62L-APC. Cells were analyzed by flow cytometry. (A) Naringenin treatment reduced the percentages of CD4^+^ T cells. (B) By other hand, splenic absolute numbers of CD3^+^ T cells from mice under Naringenin treatment were significantly reduced. (C) Representative dot plots gated on CD3^+^CD4^+^ cells showing the CD44^hi^CD62L^+^ T_cm_ and CD44^hi^CD62L^-^ T_em_ subsets. (D) Both Naringenin dose reduced the percentage of T_em_ cells and diminished (E) the splenic absolute numbers of this T-cell subset. Statistical analysis was performed by one-way ANOVA followed by Tukey´s test. Each value is the mean ± SD, n ≥ 5 mice. ** p ≤ 0.01, *** p ≤ 0.001, **** p ≤ 0.0001 compared versus vehicle.

According with previous reports, B6.MRL-Fas^lpr^/J mice exhibit an enlarged spleen as a consequence of accumulation of T cells [45,46]. Additionally, in SLE the constant presence of autoantigens inhibits the establishment of central memory (CD44^hi^CD62L^+^ T_cm_) T cells and leads to the enrichment of effector memory (CD44^hi^CD62L^-^ T_em_) pool. Then, CD4^+^ T_em_ act as IFN-γ producers and they contribute greatly in the pathogenesis of SLE [6,8]. To determine whether Naringenin inhibits the expansion of T cells in the spleen, we evaluated the percentages and total numbers of CD3^+^ and CD4^+^ T-cell subsets. Subsequently, we determinate the percentages and numbers of CD4^+^CD44^hi^CD62L^+^ T_cm_ and CD4^+^ CD44^hi^CD62L^-^ T_em_ subsets. Firstly, our results showed that Naringenin did not changes the percentage of CD3^+^ cells between groups, although Naringenin treatment increased the percentage of CD4^+^ cells, similar to cyclophosphamide, the conventional treatment against SLE (Fig 4A). By other hand, Naringenin oral administration induced a reduction in the absolute numbers of CD3^+^ T cells; however, did not observed statistical difference in the absolute numbers of CD4^+^ T cells between groups (Fig 4B). This could be due to the lower number of total splenocytes in the lupus-prone mice under Naringenin treatments. These results correlate with the Fig 1B where we findings the amelioration of splenomegaly in the B6.MRL-Fas^lpr^/J mice supplemented with Naringenin.

Naringenin administration produced changes in the percentage and numbers of splenic CD4^+^CD44^hi^CD62L^+^ T_cm_ and CD4^+^CD44^hi^CD62L^-^ T_em_ subsets. We observed an important increase in the percentage of CD4^+^CD44^hi^CD62L^+^ T_cm_. The most remarkable finding was that B6.MRL-Fas^lpr^/J mice treated with Naringenin presented a significant reduction in the percentage and absolute numbers of CD4^+^CD44^hi^CD62L^-^ T_em_ subset compared with vehicle (Fig 4C–4E).

### Oral administration of Naringenin prevented kidney damage in MRL/MpJ-Fas^lpr^/J, an aggressive model of lupus

**Fig 5:**
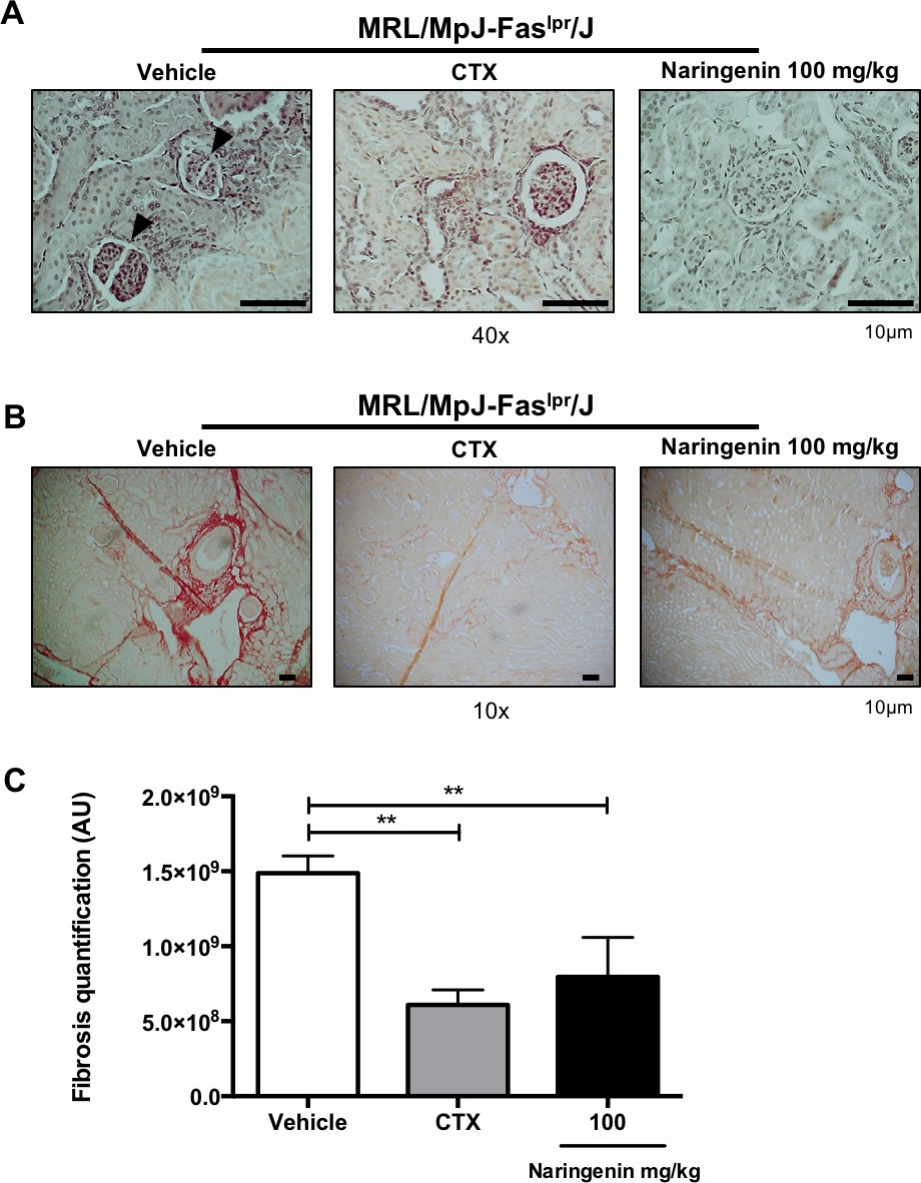
Naringenin treatment prevented kidney damage in MRL/MpJ-Fas^lpr^/J mice. Twelve-week-old MRL/MpJ-Fas^lpr^/J mice were orally administered with Naringenin at 100 mg/kg every day for six weeks. Kidneys from each mouse were removed, paraffin embedded, sectioned and stained with H&E and PSR. (A) Mice under Naringenin treatment showed less kidney damage compared with vehicle group. Representative images from H&E-stained kidney sections. The arrows indicate the damage Bowman´s capsule. (B) Kidneys from mice treated with Naringenin and cyclophosphamide presented less fibrosis. Representative images from PSR -stained kidney sections (red show fibrosis). (C) Quantification of kidney fibrosis was performed using Image-ProPlus software. Data are presented as mean ± SD and statistical analysis was performed by one-way ANOVA followed by Tukey´s test. n = 3. ** p≤ 0.01 compared versus vehicle.

Similar to patients with SLE, MRL/MpJ-Fas^lpr^/J mice present renal involvement, one of the most common complications of the disease [38,47]. In order to determinate whether Naringenin treatment prevents kidney damage 12-week-old MRL/MpJ-Fas^lpr^/J mice were administered with Naringenin at 100 mg/kg for six weeks. Similar to B6.MRL-Fas^lpr^/J mice, we provided carboxymethyl cellulose at 0.5% as vehicle and cyclophosphamide as conventional treatment. To assess renal damage, an expert pathologist evaluated histological changes in kidneys by H&E and PSR stains. The histological findings from the MRL/MpJ-Fas^lpr^/J mice provided only with vehicle showed degenerated glomerulus and broken Bowman´s capsule. Conversely, kidney sections from MRL/MpJ-Fas^lpr^/J mice treated with Naringenin did not present severe glomerular damage, similar to cyclophosphamide group (Fig 5A). Moreover, histological analysis by PSR staining revealed that kidney sections from vehicle group showed a marked increase in collagen fibers. The extent of kidney fibrosis exhibited a clear blockage in MRL/MpJ-Fas^lpr^/J mice supplemented with Naringenin (Fig 5B). Quantification of kidney fibrosis using analysis software indicated that Naringenin and cyclophosphamide treatments significantly attenuated renal fibrosis (Fig 5C).

### Naringenin treatment increased the percentage of regulatory T cells in MRL/MpJ-Fas^lpr^/J

**Fig 6:**
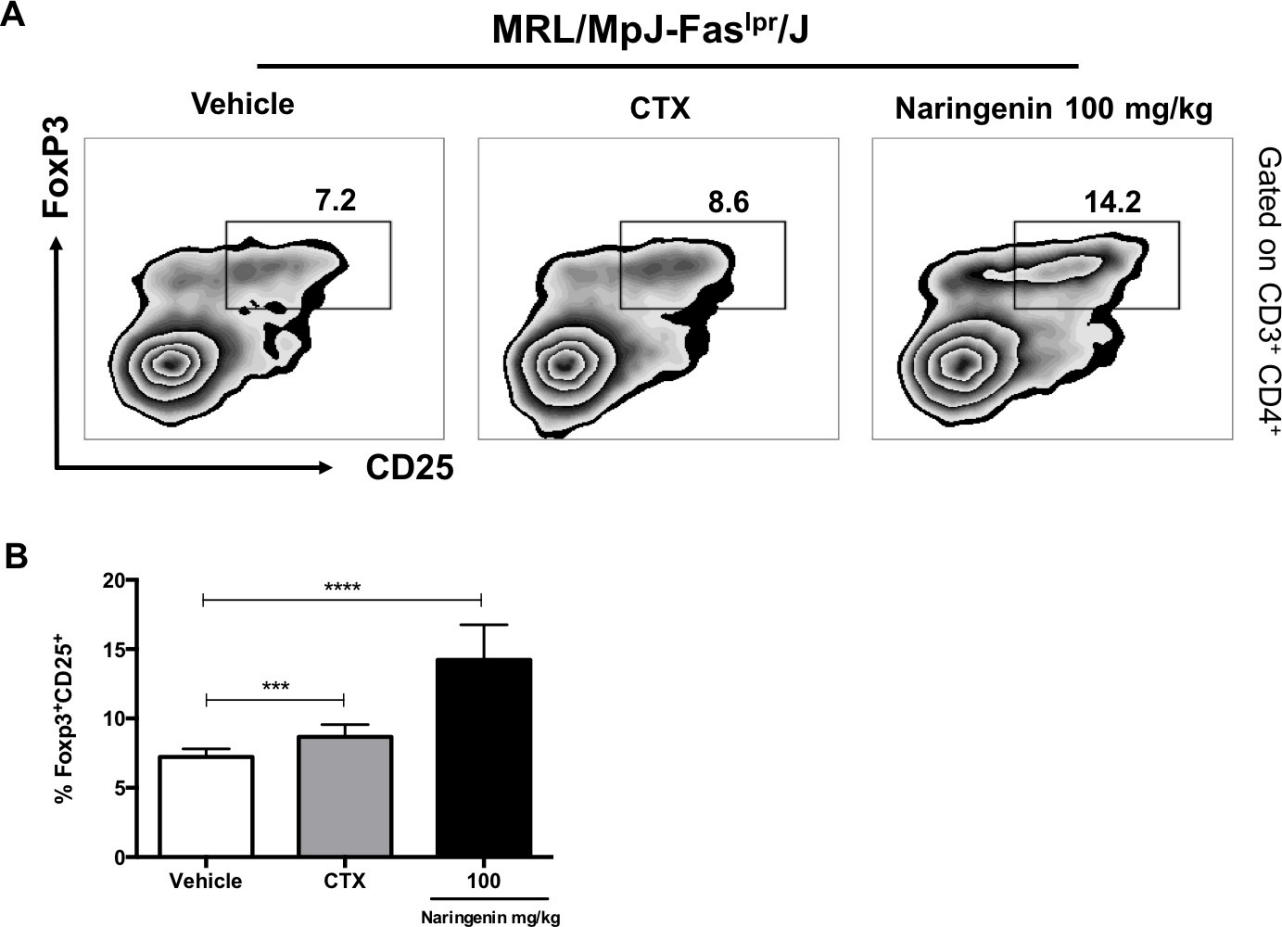
Naringenin increased the percentage of Tregs in MRL/MpJ-Fas^lpr^/J mice. Twelve-week-old MRL/MpJ-Fas^lpr^/J mice were orally administered with Naringenin at 100 mg/kg every day for six weeks. Total splenic cells were stained with anti-CD3-BV421, anti-CD4-APC, anti-CD25-APC-Cy7 and anti-FoxP3-Alexa488 as described in Materials and methods. Subsequently the cells were analyzed by flow cytometry. (A) Naringenin oral administration increased the percentage of Tregs compared with vehicle. Representative dot plots from MRL/MpJ-Fas^lpr^/J mice under the indicated treatments. (B) Percentage of CD4^+^CD25^+^FoxP3 Tregs. Statistical analysis was performed using one-way ANOVA by Tukey´s test. Data are presented as mean ± SD, n = 5. ***p≤ 0.001 compared versus vehicle.

Tregs are crucial for suppressing the immune response and self-tolerance. Altered percentages or decreased suppressive activity of Tregs has been associated with lupus pathogenesis. To determinate the percentage of splenic Tregs, 12-week-old MRL/MpJ-Fas^lpr^/J mice were orally treated with 100 mg/kg of Naringenin daily for 6 weeks. Our data revealed a significant increase in the percentage of CD4^+^CD25^+^FoxP3^+^ Treg cells in mice treated with Naringenin (Fig 6A and 6B).

## Discussion

SLE is a chronic inflammatory disease characterized by various clinical manifestations. Current therapies involve immunosuppressive and anti-inflammatory drugs that produce several side effects [21,48–50]. In this sense, the use of flavonoids as alternative therapies has increased.

Naringenin is a flavonoid has been shown to posses several biological effects including anti-inflammatory, anti-cancer and modulation of the immune system [28,30,51]. However, the effect of Naringenin on SLE remains unknown.

In this study, compared to treatment periods previously reported for other flavonoids [34,35,52], we evaluated the long-term effect (seven months of treatment) of oral administration of Naringenin on the development of autoimmunity in B6.MRL-Fas^lpr^/J lupus-prone mice. B6.MRL-Fas^lpr^/J mice resembles SLE in humans with production of several autoantibodies, imbalance in serum concentration of cytokines and abnormalities in lymphocytes subsets. However, B6.MRL-Fas^lpr^/J mice do not develop severe kidney damage compare with MRL/MpJ-Fas^lpr^/J mice, an accelerated model of SLE [38]. For this reason, MRL/MpJ-Fas^lpr^/J mice were administered with the highest dose of Naringenin, which showed the best effect in the control of SLE serological manifestations in B6.MRL-Fas^lpr^/J.

Firstly, we evaluated the effect of Naringenin on the percentage of body weight. The results showed a statistical significant difference between Naringenin treatment and the vehicle group. Both doses of Naringenin mitigated the autoimmune-prone weight loss in B6.MRL-Fas^lpr^/J mice. The cyclophosphamide group showed a similar weight loss as vehicle. This result is similar to that reported for the oral supplementation of isoflavones in lupus-prone mice, where flavonoids mitigate weight loss and the conventional treatment does not [53]. Even, Naringenin oral administration has shown to prevents weight loss in dextran sulfate sodium-induced colitis [27,28,33]. Additionally, we did not observed changes in the body weight of wild type mice supplemented with Naringenin.

In B6.MRL-Fas^lpr^/J mice splenomegaly is a classic feature of SLE and is closely related to an accelerated lymphoproliferation. Previous reports have shown that oral supplementation of Astilbin (20, 40 mg/kg in MRL/MpJ-Fas^lpr^/J mice) or Fisetin (25, 50 or 100 mg/kg in pristine-induced SLE) and intraperitoneal injection of Baicalin (200 mg/kg MRL/MpJ-Fas^lpr^/J mice) prevent the enlargement of the spleen [34,35,52]. Our results demonstrated that both doses of Naringenin ameliorated the development of splenomegaly through the reduction in accumulation of lymphocytes, which might be explained by the inhibition or reduction of proliferation, an effect previously reported for Naringenin on in vitro assays without affect the cell viability [31,32]. Moreover, we did not observed changes in the characteristics of the spleens from wild type mice provided with 100 mg/kg of Naringenin. These findings suggest that Naringenin oral administration attenuated the weight loss and moderate the lymphoproliferative disorder in B6.MRL-Fas^lpr^/J mice.

Therefore, we hypothesize that Naringenin may mitigate the main clinical features of SLE. Proteinuria reflect the grade of lupus nephritis and constitutes one biomarker for disease monitoring [41]. Although it has been reported that the administration of flavonoids such as Baicalin or isoflavones for a short period reduces proteinuria [35,53], the long-term effect of Naringenin or other flavonoids in the urine protein level has been poorly evaluated [52]. Here, we demonstrated that the highest dose Naringenin decreased the proteinuria score from the first to the third month of treatment. Despite this reduction, after the fourth month of treatment all mice presented the same proteinuria score. Interestingly, similar to our results, oral diet of Indole-3-carbinol (9 months of treatment) or Resveratrol (7 months of treatment), which are other natural compounds, decreased the urine protein levels in NZB/W F1(lupus-prone mice) or pristine-induce SLE model, respectively [54,55].

By other hand, the presence of ANAs and anti-dsDNA are closely associated with pathogenesis of lupus. About 95% of SLE patients are positive for ANAs and between 70–98% for anti-dsDNA antibodies. Both are serological markers for monitoring the disease activity [43,56]. In this regard, we measured the serum levels of these autoantibodies. Here, we indicated that the B6.MRL-Fas^lpr^/J mice treated with Naringenin showed a significant reduction in the anti-nuclear antibodies′ levels and anti-dsDNA antibodies titers compared with the mice from the vehicle group. These results correlate with previous reports which suggest that treatment with flavonoids diminish the serum concentration of autoantibodies in mice models susceptible to develop a lupus-like syndrome [34,35,53]. Therefore, the reduction of urine protein levels in the B6.MRL-Fas^lpr^/J mice treated with Naringenin could be a consequence of a lower production of autoantibodies.

In SLE patients and lupus-prone mice models, high levels of proinflammatory cytokines such as TNF-α, IFN-γ and IL-6 are present during active lupus [44,57]. For example, TNF-α and IFN-γ production correlate with the pathogenesis of SLE. It has shown that MRL/lpr mice lacking of IFN-γ receptor did not develop lupus nephritis. Also, IFN-γ induce the production of IL-6 [9,58,59]. Otherwise, IL-6 together TNF-α promotes the differentiation of B lymphocytes to plasma cells and contributes with the production of autoantibodies, particularly, anti-DNA antibodies [60]. Together, these cytokines promote glomerular inflammation and lead to renal damage. By other hand, it has been reported that IL-10 inhibit IFN-γ production, further IL-10 has a suppressive activity on the function of several immune cells. Therefore, IL-10 plays a key role in the regulation of inflammation and tissue injury [61,62]. In this sense, based on the contribution of cytokines in SLE and previous reports that suggest that flavonoids change the serum concentrations of proinflammatory and anti-inflammatory cytokines [29,34,63], we measured the serum concentration of TNF-α, INF-γ, IL-6 and IL-10. The results showed that Naringenin treatment reduced the serum concentration of proinflammatory cytokines. Then, the lower concentration of proinflammatory cytokines leads to a low production of autoantibodies and kidney damage, which is reflected in the reduction of proteinuria in B6.MRL-Fas^lpr^/J mice oral provided with Naringenin. Interestingly, the serum concentration of IL-10 was greatly increased with the highest dose of Naringenin. Naringenin treatment might promote regulatory subsets that produce IL-10 such as Tregs to control the production of proinflammatory cytokines and inhibit the function of effector cells.

The possible mechanism by which Naringenin mitigates the autoimmunity in B6.MRL-Fas^lpr^/J mice could probably be through the decrease of activated splenic T cells. Currently, there is evidence suggesting the role of CD4^+^CD44^hi^CD62L^-^ T_em_ cells in the pathogenesis of SLE, mainly for they migratory pattern and effector functions. This correlates with the increase of the expression of CD44, an activation marker [8,64,65]. Here, we also demonstrated that Naringenin treatment reduced the CD4^+^CD44^hi^CD62L^-^ T_em_ compared with vehicle. These results suggest that Naringenin have effects in the lupus manifestations through changes in the proportion of both CD4^+^CD44^hi^CD62L^+^ T_cm_ and CD4^+^CD44^hi^CD62L^-^ T_em_ subsets associated with the pathogenesis of SLE.

Renal failure represents the greatest complication of SLE. In this sense, glomerulonephritis increases the risk of developing renal failure and death. As the kidney damage is less severe in B6.MRL-Fas^lpr^/J mice compare with MRL/MpJ-Fas^lpr^/J mice [38], we administered Naringenin (100 mg/kg) for six weeks to MRL/MpJ-Fas^lpr^/J mice. The results showed that glomerular damage was prevented in mice treated with Naringenin similar to cyclophosphamide group. Furthermore, the oral administration of Naringenin avoided the development of collagen fibers compared with vehicle. It seems that Naringenin has the ability to protect against kidney injury, this effect has also been described previously for other flavonoids administered in lupus-prone mice models [35,36,52].

At last, Tregs are critical to control the immune system response and prevent autoimmune disorders. The majority of the reports suggest that a defect or decreased numbers of Tregs contribute to the development of SLE [12,66]. The suppressive function of Tregs is mediated by the secretion of anti-inflammatory cytokines such as IL-10 and TGF-β or through cell-cell contact [13]. As noted in Fig 3D, the highest dose of Naringenin increased the serum concentration of IL-10. For this reason, we also determined the percentage of Tregs in MRL/MpJ-Fas^lpr^/J mice after Naringenin treatment. We found an increased in the percentage of Tregs from MRL/MpJ-Fas^lpr^/J mice orally administered with Naringenin at 100 mg/kg compared with the vehicle. These data suggest that Naringenin promotes a rise in the percentage of Treg cells in MRL/MpJ-Fas^lpr^/J mice. These results correlate with previous reports which have described that Naringenin promoted the increase in the percentage of Tregs *in vitro* and *in vivo* [31,33].

In summary, Naringenin treatment mitigates the main characteristics of autoimmunity in lupus-prone mice through the modulation of splenic T-cell subsets and cytokines profile. Despite more studies are necessary for the full understanding of the cellular and molecular role of the effect of Naringenin in the SLE, Naringenin could be use for the treatment SLE as a complementary or alternative therapy.

## Supporting information

S1 Fig Naringenin did not affect the features of the spleens from C57BL/6J mice. Five-month-old C57BL/6J mice were oral administered by gavage daily with 100 mg/kg of Naringenin for seven months. (A) Mice treated with Naringenin did not presented change in the percentage of body weight. (B) Naringenin treatment did not change the characteristics of the spleen. Representative spleens from C57BL/6J mice from vehicle and Naringenin groups. (C) The length presented in cm. (D) The weight presented in mg. (E) Oral administration with Naringenin did not change total splenocytes number compared with vehicle. Statistical analysis was performed by t-Student´s followed by Tukey´s test. Data presented as mean ± SD, n = 5 mice. ns = no significant.
(TIF)

S2 Fig Proteinuria score previous to Naringenin treatment and from to the fourth to the seventh month in B6.MRL-Fas^lpr^/J mice. Proteinuria was semi-quantitative evaluated by test strips every month. (A) Proteinuria score before to start the flavonoid administration. (B-E) Proteinuria score from the fourth to seventh month of treatment. Statistical analysis was performed by one-way ANOVA followed by Tukey´s test. n ≥ 5. ns = no significant.
(TIF)
