# Supplementary figures and images for: Naringenin mitigates autoimmune features in lupus-prone mice by modulation of T-cell subsets and cytokines profile
Source: PLoS One. 2020 May 18;15(5):e0233138. doi: 10.1371/journal.pone.0233138 (PMC7233587; doi:10.1371/journal.pone.0233138)

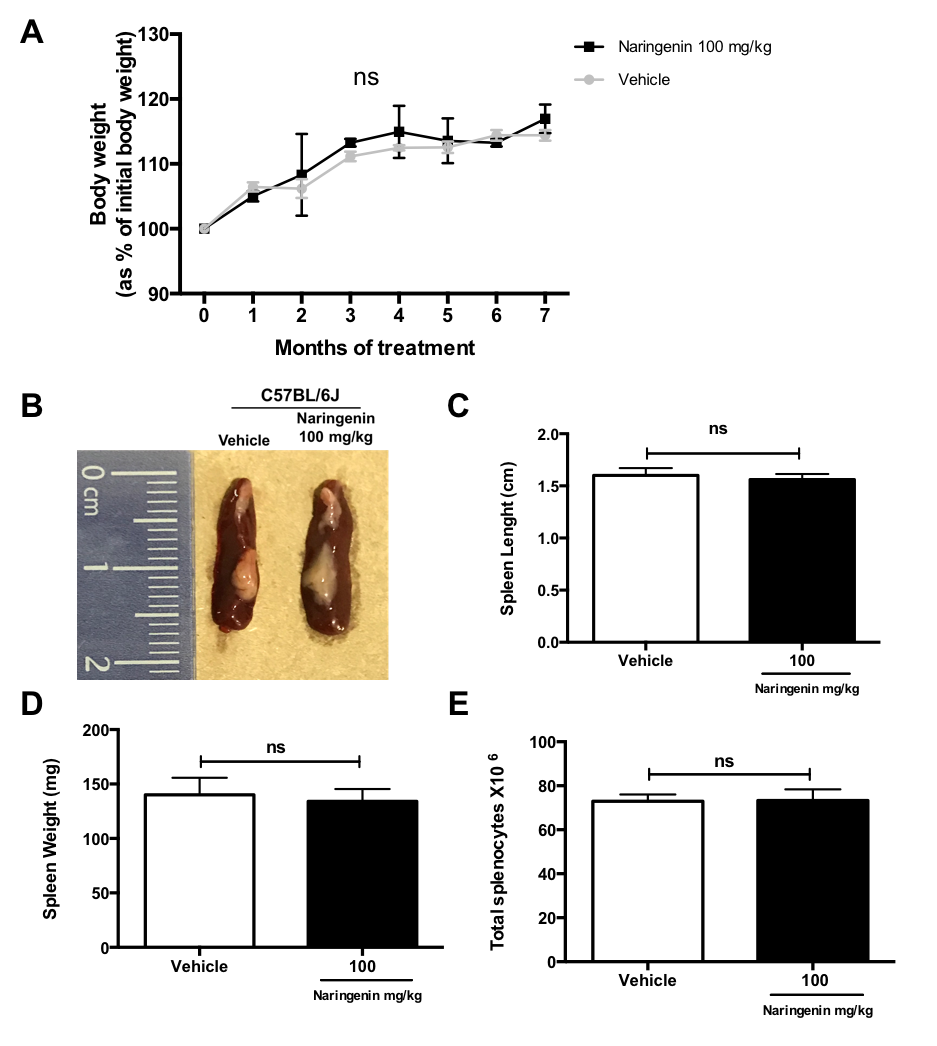

Supplement: S1 Fig — Five-month-old C57BL/6J mice were oral administered by gavage daily with 100 mg/kg of Naringenin for seven months. (A) Mice treated with Naringenin did not presented change in the percentage of body weight. (B) Naringenin treatment did not change the characteristics of the spleen. Representative spleens from C57BL/6J mice from vehicle and Naringenin groups. (C) The length presented in cm. (D) The weight presented in mg. (E) Oral administration with Naringenin did not change total splenocytes number compared with vehicle. Statistical analysis was performed by t-Student´s followed by Tukey´s test. Data presented as mean ± SD, n = 5 mice. ns = no significant. (TIF) [file pone.0233138.s001.tif]

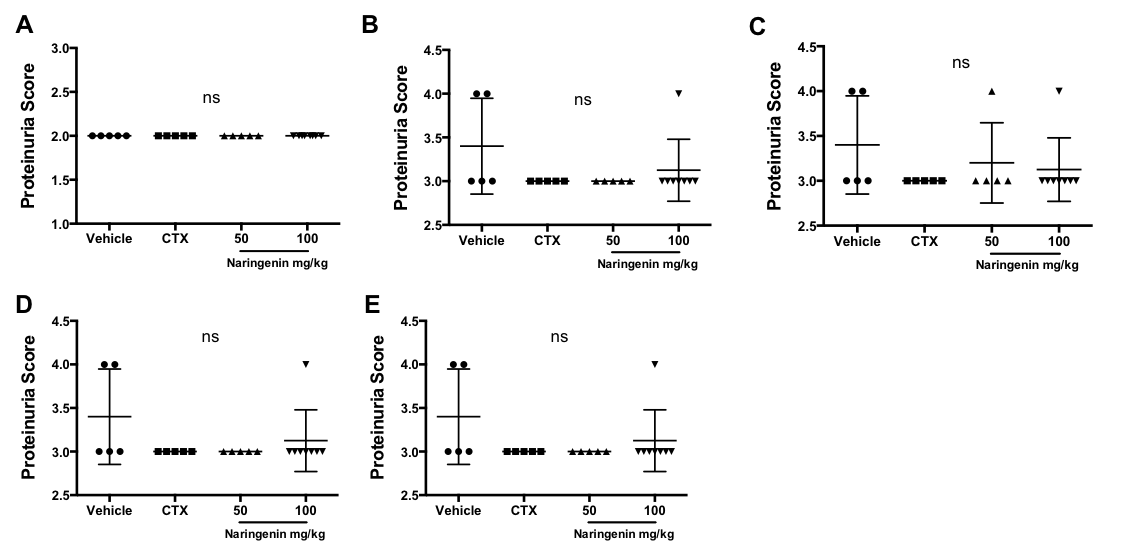

Supplement: S2 Fig — Proteinuria was semi-quantitative evaluated by test strips every month. (A) Proteinuria score before to start the flavonoid administration. (B-E) Proteinuria score from the fourth to seventh month of treatment. Statistical analysis was performed by one-way ANOVA followed by Tukey´s test. n ≥ 5. ns = no significant. (TIF) [file pone.0233138.s002.tif]
